# MicroRNAs in Honey Bee Caste Determination

**DOI:** 10.1038/srep18794

**Published:** 2016-01-07

**Authors:** Regan Ashby, Sylvain Forêt, Iain Searle, Ryszard Maleszka

**Affiliations:** 1 Research School of Biology, Australian National University, ACT, 0200, Australia; 2 Centre for Research in Therapeutic Solutions, Faculty of Education, Science, Technology and Mathematics, University of Canberra, ACT, 2617, Australia; 3 School of Biological Sciences, University of Adelaide, SA, 5005, Australia.; 4 School of Agriculture, Food and Wine,University of Adelaide, SA, 5005, Australia.; 5 The University of Adelaide and Shanghai Jiao Tong University Joint International Laboratory for Agriculture and Health, University of Adelaide, SA, 5005, Australia.

## Abstract

The cellular mechanisms employed by some organisms to produce contrasting morphological and reproductive phenotypes from the same genome remains one of the key unresolved issues in biology. Honeybees (*Apis mellifera*) use differential feeding and a haplodiploid sex determination system to generate three distinct organismal outcomes from the same genome. Here we investigate the honeybee female and male caste-specific microRNA and transcriptomic molecular signatures during a critical time of larval development. Both previously undetected and novel miRNAs have been discovered, expanding the inventory of these genomic regulators in invertebrates. We show significant differences in the microRNA and transcriptional profiles of diploid females relative to haploid drone males as well as between reproductively distinct females (queens and workers). Queens and drones show gene enrichment in physio-metabolic pathways, whereas workers show enrichment in processes associated with neuronal development, cell signalling and caste biased structural differences. Interestingly, predicted miRNA targets are primarily associated with non-physio-metabolic genes, especially neuronal targets, suggesting a mechanistic disjunction from DNA methylation that regulates physio-metabolic processes. Accordingly, miRNA targets are under-represented in methylated genes. Our data show how a common set of genetic elements are differentially harnessed by an organism, which may provide the remarkable level of developmental flexibility required.

In honeybees, three contrasting adult phenotypes are produced from the same genome as a result of biological constraints that are manifested during development^1^,^2^,^3^,^4^. Two identical diploid embryos can develop either into a functionally sterile and short-lived female worker or into a highly reproductive and long-lived queen, depending on dietary intake during postembryonic development. In contrast, male drones develop from unfertilised haploid eggs laid by a queen into special cells. Although sex determination is genetically controlled^5^, drone larvae receive a distinct diet in larger quantities compared to that of worker larvae. This suggests that similarly to queens, nutrition provides important cues for their proper development^4^,^6^.

From a developmental perspective the phenomenon of conditional phenotypes is not trivial. It requires hierarchical global reprogramming of gene expression patterns related to reproduction, longevity, cell differentiation, organogenesis, apoptosis, contrasting behaviours, etc. The flexibility of the underlying processes is underscored by recent findings that the queen phenotype can be induced not only by feeding selected larvae with royal jelly, but also by other means^7^, notably by silencing *de novo* methylation in newly hatched larvae^8^ and by interfering with the expression of genes belonging to the insulin pathway^9^.

To gain a greater understanding of the regulatory processes underlying caste specific phenotypic development in response to environmental cues, recent studies have investigated differences in the transcriptional landscape of the two female diploids to try and elucidate possible caste-biased gene expression profiles^10^,^11^,^12^,^13^,^14^,^15^,^16^,^17^. In general, larvae destined to be queens show enrichment in gene transcription associated with physio-metabolic pathways and ovary development, most likely associated with greater mass and reproductive capabilities of the larger fertile queen. In contrast, larvae destined to become the functionally smaller sterile workers show enrichment in genes associated with developmental maturation and caste biased structures such as enlarged mushroom bodies, supressed ovary development, as well as structural differences in the legs and wings required for foraging. The transcriptional profile of both female diploids varies somewhat from the above generalisations at earlier stages of larval development (L1–L3)^13^, which precede the differential expression of juvenile hormone, a process believed to be critical to the general body growth and ovary development of queens^11^,^18^. In contrast to the considerable analysis of differences in the transcriptional profile of diploid females, virtually no work has been undertaken on the transcriptional profile of the developing haploid male, a critical question addressed in the current article.

How such distinctly different transcriptional profiles are established during larval development, and how such profiles lead to caste specific phenotypic outputs, is not well understood, but appears to involve discrete changes in hormone signalling associated with dietary intake^8^. At the molecular level, the contrasting genome-wide methylation profiles in queens and workers^17^ are essential for controlling the caste-specific gene expression patterns, and in particular the patterns of alternative splicing in response to dietary cues. The role of other epigenomic modifiers in bee development is less clear, but some unusual fatty acid components of royal jelly have already been implicated in such a role^4^,^19^. They exhibit powerful histone deacetylase inhibitory activities, which results in open chromatin conformation affecting the expression of hundreds of genes from the very moment every young larva gets a mouthful of royal jelly. This idea is supported by a recent study showing that extensive post-translational histone tail modifications occur in queen ovaries and in larval tissues^20^.

In more recent times, the role of non-coding microRNAs (miRNAs) has become an area of major interest in regards to the regulation of phenotypic and developmental plasticity. As part of a larger RNA induced silencing complex (RISC), miRNAs provide sequence-specific translational repression of target mRNA molecules. They may also be involved in RNA-directed DNA methylation, translational activation and alternative splicing^21^,^22^,^23^,^24^. Increasing evidence suggests that miRNAs confer genetic robustness by reinforcing transcriptional programs, preventing major phenotypic variation in response to environmental fluctuations [canalization, for review see^25^].

Work in several species has indicated that miRNAs are important in safeguarding the correct spatiotemporal transcriptional profile, ensuring proper tissue differentiation during the movement between developmental stages, for review see^26^. In honeybees, preliminary studies on the spatiotemporal patterning of miRNA expression in adults suggested that the miRNA landscape changes during the age-dependent switch in labour division from nurse to forager in female workers^27^,^28^,^29^,^30^,^31^,^32^,^33^. However, little work has been undertaken on the role of miRNAs in the establishment of caste specific gene-expression profiles associated with the phenotypic polymorphism observed between drones, queens and workers. This study investigates differences in the miRNA profiles observed between these three castes during a critical time window of developmental canalization, or the point of no return, and how this relates to differences in their transcriptional profile and ultimately their phenotypic output.

## Results/Discussion

### MiRNA profile varies significantly between castes

**Figure 1:**
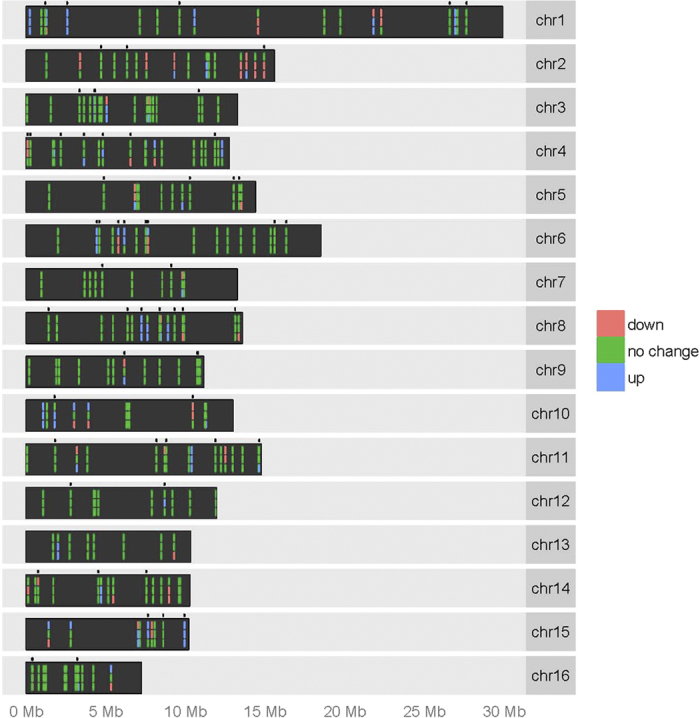
Location of miRNAs in the honeybee genome and their differential expression. For each chromosome, the top line represents miRNA expression levels in queens vs workers, middle line represents drones vs workers, and the bottom line represents drones vs queens. Green shading indicates no statistically significant difference in miRNA expression between the two castes compared, red represents a down-regulation and blue represents an up-regulation. Dots located above each chromosome indicate the location of novel miRNAs describe for the first time in this study.

In addition to the 218 known honeybee miRNAs deposited in miRBase^34^ (http://www.mirbase.org/), we identified 82 novel miRNAs in our samples (Supplementary Table S1). Novel miRNAs were dispersed throughout the genome (Fig. 1), sitting primarily within intergenic and intronic regions (Table 1). The majority of honeybee miRNAs are located in intergenic regions, but a substantial number are also found within protein coding genes, primarily in introns (Table 1). MiRNAs overlapping with protein coding genes tend to be found on the same strand suggesting that these miRNAs could be derived from mRNAs e.g. mirtrons^35^,^36^. A map of the chromosomal location of the identified miRNAs is shown on Fig. 1. MicroRNAs, both known and novel, are dispersed throughout the genome, with only a small proportion clustered in regions of 1Kb or less (Supplementary Figure S1)

After filtering out miRNAs with low counts (see methods), the expression of 164 distinct miRNAs were detected. Of these, 119 represent known or predicted sequences that have been previously deposited in miRBase, while 45 represent previously unknown sequences. The majority of the unknown miRNAs share little sequence similarity with known miRNAs from other species and therefore appear to represent miRNAs unique to *Apis mellifera*.

**Figure 2:**
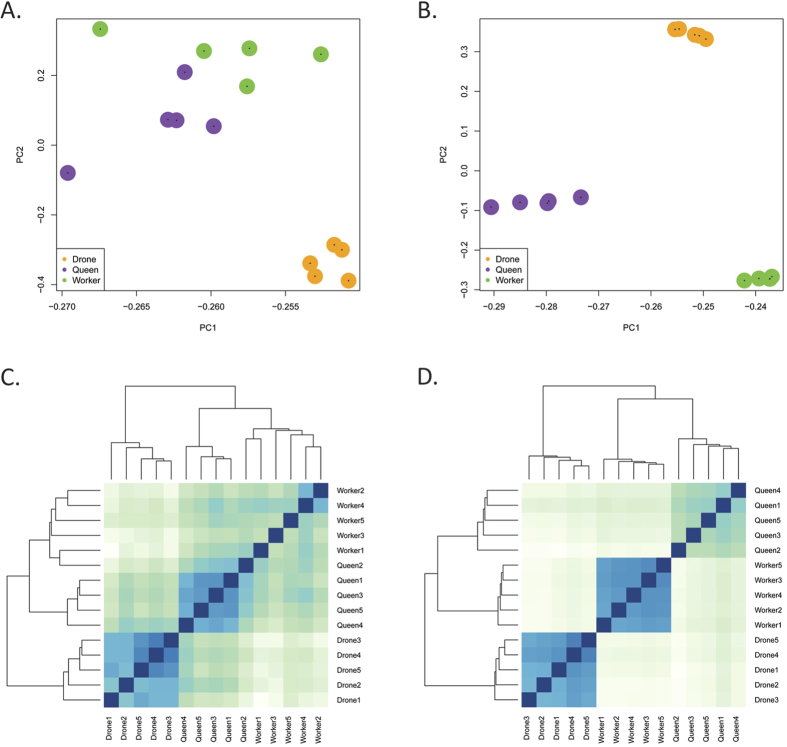
Principal component analysis of the miRNA (A) and mRNA (B) samples. Clustering using the Euclidian distance between the miRNA (**C**) and mRNA (**D**) expression vectors. The logarithm of the expression values was used.

**Figure 3:**
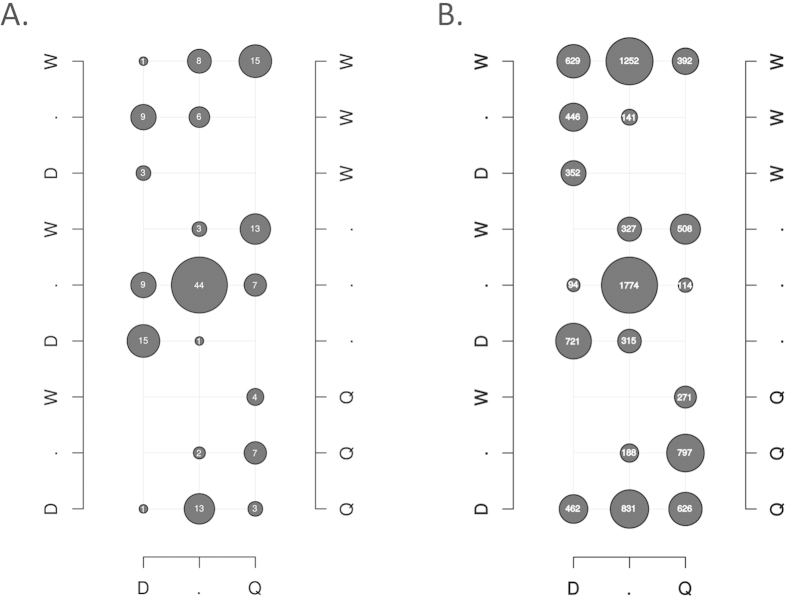
Summary of the number of differentially expressed miRNAs (A) and mRNA transcripts (B) between castes. The bottom, left and right axes represent the differences between drones and queens, drones and workers, queens and workers respectively. The letters D, Q, W indicate higher expression in drones, queens and workers respectively, while “.” denotes no differential expression. For example, when comparing the expression of miRNAs between workers and queens in (**A**), there are 42 miRNAs up-regulated in workers, 30 up-regulated in queens, and 92 miRNAs that show no change between these two castes when analysing all 164 miRNAs that were detected. For workers, the 42 miRNAs that show increased expression are displayed across the three “W” rows from right to left (15 + 8 + 1 + 6 + 9 + 3 = 42). For queens, the 30 miRNAs that show increased expression are displayed across the three “Q” rows from right to left (4 + 7 + 2 + 3 + 13 + 1 = 30), while those miRNA that show no change in expression between these two castes are displayed across the three “.” rows from right to left (13 + 3 + 7 + 44 + 9 + 1 + 15 = 92). Individual numbers represents the expression profile of that grouping of miRNAs when looking across all three comparisons (drones and queens, drones and workers, queens and workers), e.g. the top right most grouping of 15 miRNAs was up-regulated in queens relative to drones, in workers relative to drones, and in workers relative to queens.

At larval stage L4/L5, the miRNA profile is clearly distinct for each of the three honeybee castes. Principle component analysis (Fig. 2A) and clustering analysis according to the Euclidian distance between gene expression vectors (Fig. 2C) illustrates that each caste groups in a distinctly separate manner. Of the 164 miRNAs detected, 120 show differential expression between at least two castes, with 27 showing differential expression amongst all three castes (Fig. 3A). Between diploid castes, 72 miRNAs show differential expression. Of these, 42 are up-regulated and 30 are down-regulated in workers relative to queen larvae. A comparison of queens relative to the haploid drone illustrates the differential expression of 87 miRNA (49 up-regulated and 38 down-regulated). Finally, the differential expression of 80 miRNAs (44 up-regulated, 36 down-regulated) is observed in workers relative to drones. Significant overlap is seen in the expression differences observed between drones relative to either of the female castes. This suggests that the haploid system, at this developmental stage, shows clear differences in its non-coding RNA profile to that of the diploid system.

### High success rates of PCR validation confirms differential expression of miRNAs

The expression profile of 17 candidate miRNAs was further validated by StemLoop PCR (Table 2). Candidate miRNAs were chosen based on having; 1) a moderate to high expression level, 2) a differential expression with a false discovery rate of less than 5%, and 3) a fold change of more than 1.5 in at least one of the three comparisons between castes. A between-caste comparison of the sequencing data indicated 36 differential changes in the expression of the 17 candidate miRNAs chosen. In the majority of cases, differential expression represented a reduction in miRNA levels in one or both of the diploid females relative to drones (this pattern represents 20/36 changes observed). Of these changes, 31 were validated by StemLoop PCR (Table 2), representing an 86% validation rate. Of the 15 conditions in which the sequencing data indicated no differential expression should be observed, 13 were validated by PCR (Table 2), again resulting in a high validation rate of 87%.

### Transcriptional profile of the honeybee at larval stage L4/L5

At this larval stage (L4/L5), when the commitment to a specific developmental trajectory is virtually irreversible, the transcriptional profile of the three honeybee castes is clearly distinct. Principal component analysis (Fig. 2B) and clustering according to the Euclidian distance between gene expression vectors (Fig. 2D) show each caste grouping separately.

### Gene Ontology (GO) and pathway analysis substantiate phenotypic distinctiveness of female castes

Distinct differences in the gene enrichment profile are observed between all three castes at 96hrs of larval development, as determined by both Gene Ontology (GO) and pathway analysis (using the Kyoto Encyclopaedia of Genes and Genomes, KEGG). Queen larvae show gene enrichment in pathways associated with physiometabolic processes (Supplementary Table S2, sections 1.1 and 1.2), especially in the catabolism of the three major classes of macromolecules (proteins, carbohydrates and fats) as well as the biosynthesis of juvenile hormone (JH, Table 3), consistent with the significant increase in JH levels in queens at this developmental stage^11^. This is indicative of the increased growth rates seen in queen larvae during this period. In our bee colonies, 96 hr-old queen larvae have a body weight ranging from 200–310 mg, whereas the weight of worker larvae is typically 170–200 mg. For comparison, drone larvae of similar age can reach body mass of up to 400 mg. The largest changes are apparent in pathways associated with protein metabolism followed by a significant up-regulation in genes associated with the major energy pathways, including; the citric acid cycle (Table 4), pentose phosphate pathway, glyoxylate metabolism, glycolysis/gluconeogenesis, oxidative phosphorylation and pyruvate metabolism. Pathways associated with carbon fixation and metabolism are also significantly up-regulated, as well as genes associated with the metabolic pathways of lysosome activity, folate biosynthesis, the metabolism of propanoate and xenobiotics, as well as mitochondrial development.

In contrast to queens, workers show a profile indicative of an increased rate of developmental maturation and caste biased structural differences (Supplementary Table S2, sections 1.1 and 1.2). Strong enrichment is seen in genes associated with the major cell signalling pathways involved in embryonic development, including hippo, Wnt, MAPK/ERK, hedgehog, PI3K/AKT, Notch, VEGF and calcium signalling. In diploids, a number of these pathways also show differential methylation, especially PI3K/AKT^17^. Workers also show enrichment in genes associated with cell guidance (chemokine signalling), adhesion and microfilament formation (e.g. actin cytoskeleton), as well as pathways associated with transcription, translation and protein processing. During the fourth and fifth stage of larval development, workers show a reduction in ovariole numbers, through programmed cell death. Consistent with this observation, the associated apoptotic pathways show gene enrichment (e.g. β-catenin), similar to that reported previously^11^,^13^,^16^,^37^.

Workers also show significant enrichment in neuronal pathways, including; cholinergic (Table 5), glutamatergic and dopaminergic synaptic development, axon guidance, retinal phototransduction, ommatidial rotation, olfactory learning, synaptic vesicle formation, peripheral nervous system development, potassium and calcium ion transmembrane transport and voltage-gated potassium channel complex formation. This distinctiveness in neuronal development/maturation in the worker larvae may be associated with the early stages of building a sophisticated nervous system required for their remarkable navigational skills and high mnemonic fidelity during adult life. Workers also show caste specific structural differences in the morphogenesis of both their legs and wings to allow them to undertake foraging tasks (e.g. unique structures for pollen and propolis collection). This is underscored by enrichment in genes associated with imaginal disc-derived leg morphogenesis, wing disc dorsal/ventral pattern formation and morphogenesis, and positive regulation of synaptic growth at neuromuscular junctions during this critical period of caste determination. Relative to queens, workers also show gene enrichment in pathways associated with; digestive activities (gastric acid secretion), hormone biogenesis (insulin and aldosterone), melanogenesis (believed to be critical for innate immunity)^38^, and circadian entrainment.

### GO and pathway analysis reveal contrasting regulation in haploid males versus diploid females

Relative to workers, drones show a gene enrichment profile similar to queens, with an up-regulation in genes associated with physio-metabolic processes (Supplementary Table S2, sections 1.1 and 1.2). Relative to drones, workers again show enrichment in genes associated with developmental maturation, cell signalling, neuronal maturation and caste biased structural differences.

Interestingly, drones show an even greater level of gene enrichment in a number of physio-metabolic components relative to queens, indicating a possible higher rate of metabolic activity (Supplementary Table S2, section 1.1 and 1.2). Drones also show increased expression of genes associated with the metabolism of proteins, carbohydrates, carbon and methane, as well as structural constituents of ribosomes and cuticles. In contrast, queens show gene enrichment in pathways associated primarily with oxidative phosphorylation and protein processing and exportation relative to drones.

### Predicted miRNA targets are part of distinct gene pathways

MicroRNA target prediction was initially undertaken using a minimum of two out of three *in-silico* prediction methods (see methods). From this initial analysis, the ontology terms (biological processes) that showed the greatest enrichment for predicted targets included; transcription factor activity, steroid hormone pathway, neuronal development (brain morphogenesis, olfactory learning and axon guidance/formation), ion channel formation (specifically potassium voltage-gated and glutamate-gated), and imaginal disc-derived wing morphogenesis (Supplementary Table S2, section 2.1).

**Figure 4:**
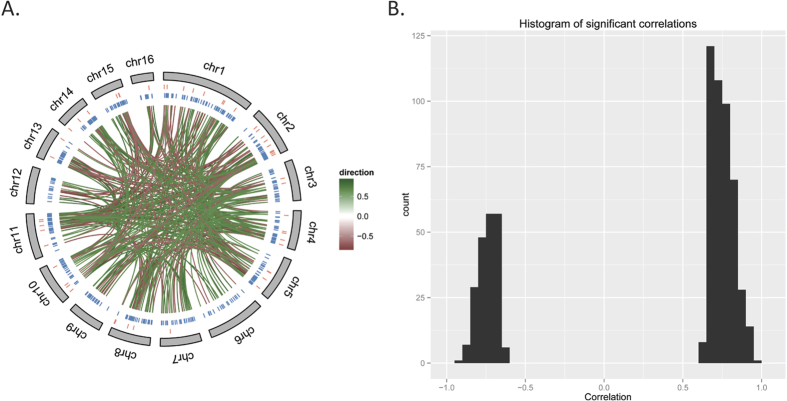
Chromosomal distribution of miRNAs and their predicted mRNA target genes. (**A**) Genome-wide view of the statistically significant correlations between miRNAs and their predicted targets. The outer circle represents the chromosomes, the next inner track represents the location of the miRNAs, followed by their targets and the links between the two. The links are coloured according to the value of the Pearson correlation coefficient. (**B**) Distribution of statistically significant Pearson correlation coefficients between miRNAs and the mRNA level of their target genes, indicating that more than half of the correlations are positive. A positive correlation indicates that the direct of change observed in the expression of the miRNA and its target transcript was the same. In contrast, a negative correlation indicates the change in expression of the miRNA and its target was in the opposite direction.

This list was further refined by accepting only miRNA target pairs with a statistically significant Pearson correlation coefficient (Fig. 4A). Based on this analysis, 654 interactions were predicted to occur, involving 96 miRNAs and 526 target transcripts (Supplementary Table S3), 87% of which are differentially expressed, supporting the validity of the predicted targets. Of the predicted interactions, 449 involve a positive correlation, in which both the miRNA and the targeted transcript show a similar direction of change, while 205 show a negative correlation (Fig. 4B). A number of miRNAs are predicted to target multiple genes, the maximum being ame-mir-210 with 68 interactions. Similarly, many transcripts are targeted by more than one miRNA, with the transcription factor mblk-1 (GB50048) showing the largest number of predicted interactions (seven). Mblk-1 is a transcription factor expressed within neuronal tissue, specifically the mushroom bodies, and appears to be modulated by the Ras/MAPK pathway^39^. Both neuronal tissue and cell signalling pathways such as Ras/MAPK appear to be primary targets of the miRNA system, as discussed below.

Of the 526 predicted transcripts that show a correlation with their targeting miRNA, 371 show a positive correlation, 190 show a negative correlation, while 35 show both. A similar breakdown was seen for the 96 miRNAs involved, with 73 showing a positive correlation to their targets, 71 showing a negative correlation and 48 showing both. Interestingly, it is often assumed that a negative correlation should predominate. That is, a detectable reduction in transcript numbers would be observed following the up-regulation of the targeting miRNA, if cleavage of the target transcript occurred. However, miRNAs, for the most part, only moderately repress their targets, and appear to confer genetic robustness by preventing the overexpression of target genes during fluctuations in environmental cues, for review see^25^. Under this framework, a positive correlation would not be unexpected, as reported recently in other biological contexts^40^,^41^. For example, in response to an environmental cue such the ingestion of royal jelly, the transcriptional output of specific developmental genes may increase. However, overexpression of such developmental pathways would be deleterious to the organism and may therefore be prevented by increased miRNA expression, buffering translational output. In this scenario, although both the target transcript and miRNA would be up-regulated, the miRNA is still acting to reduce (buffer) the rate/amount of translation of the targeted gene relative to that which would occur in the absence of the miRNA. This senior could also occur when the targeting miRNA interferes with the translational machinery rather than causing target-specific mRNA cleavage (for a review of possible translational suppression methods, see^42^). In this case, transcription levels could rise or remain unchanged, yet translation levels may be suppressed.

Analysis of GO terms under the categories of biological processes and molecular function indicates that miRNAs in the honeybee are predicted to overwhelmingly target transcription factors within pathways associated with neuronal development (Supplementary Table S2, sections 2.1 and 3.1; Supplementary Table S3). Specifically, gene enrichment was seen in pathways associated with; axon guidance and development, synapse formation (target recognition, vesicle formation, synaptic growth, formation of neuromuscular junctions, dopaminergic and cholinergic synapse formation), telencephalon development, visual behaviour, neuronal specific ion channels, and learning and memory. This is consistent with the reported enrichment of miRNAs within neural tissue of many species, where they appear to play a critical role in synaptic plasticity, axon guidance, as well as learning and memory, for review see^43^,^44^,^45^. For example, mir-124 is involved in neuronal cell differentiation through a negative feedback loop with the membrane protein ephrin-B1, a member of the ephrin signalling pathway^46^. In the developing mouse brain, ephrin-B1 holds cells in their progenitor state, while mir-124 promotes their differentiation through inhibition of ephrin-B1 expression. In workers, the expression of ame-mir-124 and components of the ephrin signalling pathway (ephrin receptor tyrosine kinase (GB41629)^47^, ephrin-B2a (GB40333)^47^, ephrin protein (GB43375) and Pak1 (GB48749)) were significantly higher relative to queens, suggestive of significant neuronal maturation occurring in workers at L4/L5.

Other miRNA target pathways that show significant gene enrichment include; cell signalling (Notch, Wnt and steroid hormone signalling, as well as tyrosine kinase activity), apoptosis, modulation of transcription factor activity, and cellular differentiation/migration (chemotaxis, cell cycle arrest, cell migration, cell delamination, tube morphogenesis and anterior/posterior patterning). Pathways associated with both wing and leg imaginal disc morphogenesis also shows significant enrichment.

A number of the miRNAs found to be differentially expressed in our current study form critical components of the major cell signalling pathways in other species, including ame-Bantam, ame-mir-315 and ame-Let-7. Outside of *Drosophila*, the role of miRNAs in insect development has not been extensively studied. However, some insight can be drawn from other species, including *Drosophila*. For example, Bantam is an important target of the Hippo and EGFR/MAPK signalling pathways^48^,^49^,^50^, both critical in embryonic development and the control of cell proliferation and survival. In *Drosophila*, mir-315 expression affects the conserved Wingless (Wg or Wnt) signalling pathway^51^, which modulates tissue patterning and cell differentiation in many species. Mir-let-7 is a major target of the steroid pathways underlying temporal cell fate determination during the movement between developmental stages. Let-7 is one of the few consistent miRNA markers associated with the behavioural shift of worker bees from nurses to forages^27^,^29^,^31^. Royal jelly has been reported to contain significant quantities of ame-mir-Let-7^32^, consistent with the idea that let-7 levels are higher in queens relative to workers at this critical larval stage. In *Drosophila*, the developmental progression from larvae to pupae has been shown to involve the modulation of the Let-7-C polycistronic miRNA cluster (Let-7, mir-100 and mir-125), as well as mir-8 and mir-34^52^, by the lipophilic hormones 20-hydoxy ecdysone (20E) and juvenile hormone (JH). Each of these miRNAs was also observed to be differentially expressed in our current study, with Ame-mir-34 showing one of the largest sets of predicted transcript targets (20). The majority of these predicted targets are associated with neuronal development or ion channel formation. One such transcript is semaphorin 2a (GB46091). In both vertebrates and invertebrates, semaphorins are involved in axon guidance and ultimately target selection and synapse formation^53^. In *Drosophila*, semaphorin 2a acts as a chemorepulsive axon guidance cue critical for neuronal pathfinding and synaptic arborisation^54^. Semaphorin 2a (GB46091) mRNA levels were significantly elevated in workers relative to queens, further suggestive of greater levels of neuronal development and maturation occurring in workers at this critical larval stage.

As mentioned above, movement between developmental stages in both *Drosophila* and the honeybee involves the activity of the steroid hormone 20E, which acts through the ecdysone receptor (EcR). In our current study, EcR mRNA levels were elevated in workers relative to queens. EcR expression and activity levels have been shown to be negatively regulated by mir-14^55^. Consistent with this role, ame-mir-14 levels were elevated in queens relative to workers.

It is noteworthy that broadly similar GO categories were identified by another parallel analysis where instead of looking at the targets at the intersection of different miRNA target prediction software, we looked at the intersection between the GO terms predicted by each software separately (see methods and Supplementary Table S4).

### DNA methylation and miRNAs target different developmental pathways

When we considered miRNA target pairs predicted by at least two software approaches, we found that predicted targets were significantly underrepresented amongst methylated genes (Fisher exact test, p < 1 × 10^−6^). When we looked at the predicted miRNA target pairs generated by each individual software, however, this underrepresentation was only confirmed with RNAhydrid (p < 1 × 10^−21^), but not PITA (p = 0.24) nor Miranda (p = 0.14). Although this possible underrepresentation requires further analysis, DNA methylation appears to be predominantly associated with physiometabolic processes, a functional category that was not heavily targeted by miRNAs. Instead, miRNAs appear to overwhelmingly target neuronal maturation and caste biased structural differences in the honeybee. This suggests a distinct evolutionary division in the developmental processes that are regulated by DNA methylation and miRNAs.

**Figure 5:**
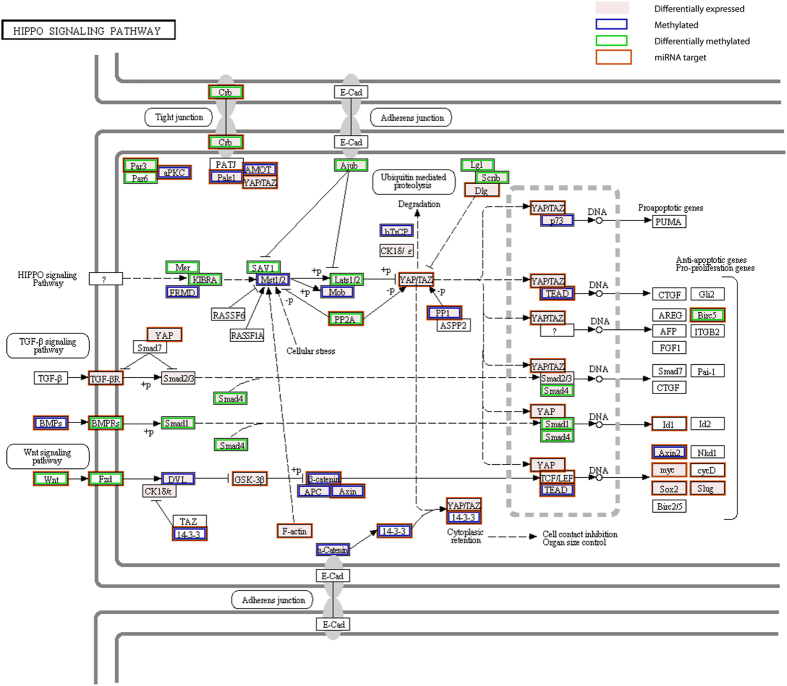
Regulation of the Hippo pathway in honeybee larvae by miRNAs and DNA methylation. The Hippo pathway plays important roles in growth and development; it is enriched in differentially expressed genes, differentially methylated genes and miRNA targets. A pink shade indicates differential expression between any of the three larval castes. A blue outline indicates DNA methylation in queen or worker larvae, a green outline denotes differential DNA methylation between these. Predicted miRNA targets are outlined in brown. Based on KEGG map04390 (http://www.genome.jp/kegg-bin/show_pathway?map04390).

Some overlap in the regulatory patterns of methylation and miRNAs was observed with respect to the major cell signalling pathways. The Hippo signalling pathway is one such striking example (Fig. 5). At the level of the whole larvae, this pathway was significantly enriched in differentially expressed genes between castes (Supplementary Table S2), many of which are predicated targets of miRNAs (Supplementary Table S3). Based on sequencing data from larval heads, this pathway is also enriched in differentially methylated genes between queens and workers (Fig. 5). The Hippo pathway plays an important role in the control of organ growth and cell proliferation^56^. Our results suggest that the development of morphological differences between castes requires a complex fine-tuning of this pathway by miRNAs and DNA methylation.

### Insights into gender-related disparity in immune systems in a social organism

It is often argued that gender-specific immune responses exist because females gain fitness through increased longevity, whereas males gain fitness by increasing mating rates. These contrasting investment strategies result in a higher susceptibility of males to pathogens and other stressors^57^. Our results add some support to the notion that females have stronger immune responses than males. For example, in both queen and worker larvae, genes encoding the antimicrobial peptides Defensin and Apidaecin belong to the most highly expressed category, whereas in males these peptides are expressed at extremely low levels. In the social context it is conceivable that drone larvae are partly shielded against stressors at the colony level by ‘social immunity’ that is provided by hygienic behaviour of adult nurse bees. These collective behaviours compensate for the smaller immune proteome in honeybees relative to solitary insects^58^. An extra level of protection for the queen larva is provided by royal jelly that has strong antimicrobial properties. The contribution of specific miRNAs to gender-associated immunologic processes in the honeybee is less straightforward because many immune-related miRNAs also regulate growth and apoptosis that are differentially manifested in the three castes. The highly conserved mir-1175 whose expression is affected by bacterial infection in adult honeybees^59^ and in mosquitos^60^ shows differential expression during larval growth across all three castes. Several other miRNAs, postulated to be involved in immune regulation^52^,^60^,^61^,^62^, are also differentially expressed in our study, including; Let-7, mir-8, mir-12, mir-33, mir-34, mir-190, mir-279, mir-283 and mir-989.

### MicroRNAs in caste determination

Our data clearly show that the expression profile of miRNAs is distinctly different between castes at this critical stage of larval development. These non-coding RNAs may therefore form an integral part of the molecular framework underlying developmental canalisation in the honeybee. The most important insight from our study is their apparent complementary role to that of DNA methylation based on miRNA target prediction. Our findings indicate that the interactions between miRNAs and their target transcripts form a highly interconnected network that appears to be biased towards regulating cellular processes involved in neuronal differentiation, cell signalling and the formation caste-specific structures. These processes are distinct from those controlled by DNA methylation, which have been shown to predominantly modulate physio-metabolic and information processing genes, whose products are known to regulate the rate of mass-transforming processes and the general growth of an organism^2^,^11^,^17^. The striking differences in both miRNAs and mRNAs profiles between queens, workers and drones occur during a critical larva-to-pupa transition when larval imaginal discs begin to differentiate into various outside body parts. The robustness of this process is essential to create the desired adult phenotypes associated with different life challenges. The streamlined behaviours of queens and drones are primarily geared towards reproduction, whereas sterile workers perform a wide range of tasks and undergo behavioural maturation that prepares them for complex hive building and foraging tasks. One possibility is that during the initial hours of larval development, a high level of transcriptional noise, resulting either from differential feeding (environment) or a haploid genome, is necessary to alter developmental trajectory. However, such alternative trajectories need to be rapidly stabilised to ensure phenotypic constancy^63^. A role of miRNAs in this context would be to buffer fluctuations in target gene expression, not only to ensure robustness of the desired phenotype, but also to supress alternative ones. On the other hand, DNA methylation is a highly flexible epigenomic modifier, responding to both internal and external stimuli. It may therefore play a role in generating alternative patterns of gene expression and in doing so act as a driver of developmental plasticity. Indeed, silencing *de novo* methylation in newly hatched larvae results in a strong bias for queen phenotype^8^.

## Conclusion

Our study provides important insights into the possible role of miRNAs in shaping the conditional phenotypes associated with male and female castes in *Apis mellifera* and the associated flexible behaviours that arise from them in response to the integration of environmental and genomic signals. The distinct differences in behaviour, physiology, longevity and reproductive capacity between castes is driven by changes in the epigenetic landscape of specific transcriptional networks in response to diverse cues. We provide evidence that miRNAs may play a significant role in this process by targeting non-methylated genes associated primarily with neuronal development and thus complementing the regulatory effects of the DNA methylation toolkit. We propose that future studies on the epigenetic plasticity of different cell types, such as larval imaginal discs and their epigenomic signatures are required to achieve the in-depth understanding of novel developmental outcomes.

## Material and Methods

### Sample Collection

Honeybee (*Apis mellifera var. ligustica*) larvae were collected from a single hive located at the Australian National University (ANU) in Canberra. To obtain specimens in similar developmental stages, newly capped queen, worker and drone larvae were removed from the frame and frozen in liquid nitrogen. Both queen and worker larvae were 96 ± 1 hr-old at the time of collection, whereas drone larvae were 96 ± 5 hr-old. This developmental phase corresponds to stage L5 after the 4^th^ molt. At this point the commitment to a particular developmental trajectory is irreversible^64^. 25 larval samples for each caste were split into five biological replicates consisting of five larvae each.

### RNA extraction

As previously described^65^, total RNA was extracted from larval samples using Trizol reagent, following the manufacturer’s protocol (Invitrogen Life Technologies, Australia). Briefly, larval samples were homogenised in 500 μl of Trizol reagent (3:1 Trizol to sample ratio). One hundred microliters of chloroform were then added and allowed to incubate for 3 min at room temperature, before the samples were centrifuged at 12,000 g for 10 min at 4 °C. Following centrifugation, the upper phase was collected, with total RNA precipitated using isopropanol. The pellet was washed briefly in 75% ethanol and dissolved in 50 μL double-distilled water by incubating for 2–3 min at 80 °C. RNA integrity was determined by gel electrophoresis and quantified using a NanoDrop spectrophotometer.

### RNA Libraries Preparation for Illumina high throughput sequencing

Briefly, small RNAs were size selected and purified from 10 μg of total RNA (n = 5 per caste) using a 15% acrylamide:bis-acrylamide PAGE, 8M urea gel with the aid of a miRNA Marker (#N2102S, NEB). The size selected small RNAs were eluted from the gel by incubation in a 0.3M NaCl solution over night at 4 °C, before precipitation using the isopropanol/ethanol. The precipitated pellet was dissolved in 6.5 μL RNAase free, DEPC-treated, distilled water. Library preparation, which involved adaptor ligation, reverse transcription and PCR amplification, were carried out following the manufactures protocol for small RNA library preparations (#E7300S/L, New England BioLabs (NEB), Genesearch, Australia). Product verification and size selection of amplified cDNA libraries was undertaken using a 6% PAGE gel. Gel bands corresponding to 140–150 nucleotides in size, which corresponds to adaptor-ligated constructs formed from 21–30 nucleotide RNA fragments, were excised from the gel and extracted by overnight incubation in 0.3M NaCl at 4 °C. Library constructs were then precipitated with 3M NaOAc, chilled isopropanol, before being washed in 75% ethanol, with the final pellet dissolved in 10 μl 10 mM Tris‐HCI, pH 8.5. Libraries were validated on a 2100 Bioanalyser (Agilent Technologies, Australia), using a high sensitivity DNA LabChip. mRNA libraries were prepared using the NEB ultra-directional kit (#E7420S). Sequencing was carried out at the Biomolecular Research Facility (John Curtin School of Medical Research, ANU, Canberra) on an Illumina HiSeq 2500 platform. Transcriptome and miRNA sequence information and raw counts have been submitted to the GEO NCBI database (accession number GSE61253).

### Validation of miRNAs by Stem-Loop PCR

Stem-Loop qRT-PCR validation of miRNA expression, using Universal ProbeLibrary Probe 21 (Roche Diagnostics Australia, Castle Hill, Victoria), was adapted from the protocol of Varkonyi-Gasic, Wu^66^. Total RNA was isolated from larval samples (n = 5 per caste) that were independent of those processed for sequencing, using the Trizol methodology detailed above. Total RNA purity was determined by gel electrophoresis and quantified using a NanoDrop spectrophotometer. Reverse transcription was performed in a 15 μl reaction volume containing; 15 ng of total RNA, 50 nM Stem-Loop RT Primer (Supplementary Table S5), which was first combined and heated to 65 °C for 5 minutes, 1x SuperScript VILO Reaction Mix (Invitrogen Life Technologies, Australia), 1x SuperScript VILO Enzyme Mix, and 20 units of RNaseOut (Invitrogen). The final reaction mixture (15μL) was heated to 16 °C for 30 minutes, 42 °C for 30 minutes, with the reaction terminated by incubating at 85 °C for 5 minutes and then cooled to 4 °C on an Eppendorf Master thermocycler.

RT-PCR was performed on a StepOnePlus Real-Time PCR System (Applied Biosystems (Life Technologies), Melgrave, Vic, Australia), using the Universal ProbeLibrary probe assay detailed in Varkonyi-Gasic, Wu^66^. The 15μL PCR reaction mixture consisted of; 1μL of cDNA, 50 nM forward and universal reverse primers (Supplementary Table S5), 10 nM Universal Probe 21, and 1xFastStart Universal Probe Master Mix (Roche Diagnostics Australia). Cycling conditions were; initial denaturation at 95 °C for 5 minutes, followed by 40 cycles consisting of; 95 °C for 10 seconds, 60 °C for 30 seconds and 72 °C for 10 seconds. Specificity of the PCR products was determined by agarose gel electrophoresis, melt curve analysis and sequencing.

### Bioinformatics analyses

Throughout this study, the honeybee genome assembly version 4.5 and the official gene set version 3.2^58^ were used.

### Prediction of novel miRNAs

Small RNA reads were first mapped to the honeybee rRNA and tRNA sequences to remove potential contamination from these transcripts. Novel miRNAs were predicted using the mirDeep2 software v2.0.0.5^67^ with default parameters, discarding reads smaller than 18bp. Predictions were run first on the 15 (5 drones, 5 queens, 5 workers) samples separately, and then on all the reads from the different samples combined together. These 16 sets of predictions were then merged and manually curated. MirDeep2 was also used to calculate the read counts of each miRNA transcripts.

### Mapping mRNA

RNA-seq reads were mapped onto the honeybee genome using the TopHat software v2.0.9^68^ with the following parameters: —read-mismatches 5 —read-gap-length 4 —read-edit-dist 6 —read-realign-edit-dist 5 —min-intron-length 40 —min-segment-intron 40 —library-type fr-firststrand —b2-very-sensitive, and using the –GTF parameter to provide the gene models of the official gene set v3.2. Counts per transcripts were calculated using the htseq-count tool from the HTSeq v0.5.4p3 package^69^ with the following parameters: —stranded =yes—mode= intersection-nonempty.

### Differential gene expression and gene set enrichment analysis

Differentially expressed miRNAs and mRNAs were identified using the EdgeR package from Bioconductor release 2.12^70^. Only the miRNAs and mRNAs with at least 5 counts in at least 5 samples were considered. Differentially expressed genes were called at a stringent false discovery rate of 1%^71^. GO annotations were carried out with blast2go^72^ and KEGG annotations with KASS^73^. Gene set enrichment analyses in GO terms or KEGG pathways were carried out with GoSeq^74^ to correct for selection bias caused by gene length using a false discovery rate of 5%.

### Prediction of miRNA targets

We searched for targets in the 3′UTR of genes models. For genes lacking a predicted ′’ UTR, 500bp downstream of the stop codon were selected. We used three different target prediction methods (PITA^75^, Miranda^76^ and RNAHybrid^77^) with a maximum free energy of -10, -20 and -27 kcal/mol respectively. As miRNA target predictions tend to have a high false positive rate, we refined our predictions by calculating the Pearson correlation coefficient between the expression levels of each miRNA and their targets as describe in^78^. Only miRNA target pairs significantly correlated with a false discovery rate lower than 5% were considered. As it has been reported that taking the intersection of different target prediction methods does not necessarily increase the true positive rate^79^,^80^, we used two different approaches to combine our target predictions. In the first approach, only targets predicted by two of the three methods were considered (Supplementary Table S3). In the second approach, we considered the predictions separately and then looked at the overlapping enriched GO categories (Supplementary Table S4).

### DNA methylation

DNA methylation analysis was carried out as described in Forêt *et al.*^17^.

### Data Access

Sequence data and raw counts have been submitted to GEO NCBI (accession number GSE61253).

## Additional Information

**How to cite this article**: Ashby, R. *et al.* MicroRNAs in Honey Bee Caste Determination. *Sci. Rep.* **6**, 18794; doi: 10.1038/srep18794 (2016).

## Supplementary Material

Supplementary Information

Supplementary Table S1

Supplementary Table S2

Supplementary Table S3

Supplementary Table S4

## Figures and Tables

**Table 1:** Genomic location of novel miRNAs.

miRNA category	Intergenic	Intronic	UTR	CDS
Known	105	85 (54:31)	8 (4:4)	20 (13:7)
Novel	42	32 (28:4)	3 (3:0)	5 (5:0)
Total	147	117 (82:35)	11 (7:4)	25 (18:7)

We identified 218 known miRNAs and 82 novel miRNAs. These miRNAs were predominately located in intergenic regions however a significant number were located in introns. For intronic, untranslated region (UTR) and coding regions (CDS), the first number in parenthesis indicates the number of miRNA on the same strand as the corresponding gene, and the second number indicates the number of the reverse strand. The known miRNAs are those already deposited in miRBase.

**Table 2:**
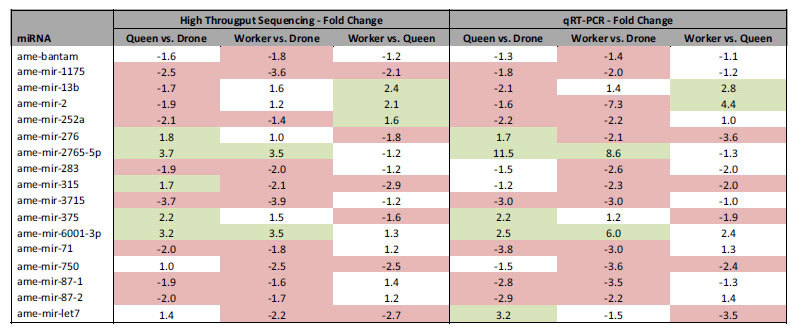
Candidate validation of miRNA expression levels from drone, queen and worker larvae by StemLoop PCR.

**Table 3:** Increased expression of enzymes assocaited with juvenile hormone (JH) biosynthesis in queens relative to workers.

Enzyme (JH Biosynthesis)	GB Number	Log Fold Change*	FDR	Function
Adenosylhomocysteinase	GB14324	−0.55	1.86×10^−16^	Hydorlysis of S-adenosylhomocysteine
Adenosine kinase	GB13063	−1.12	1.53×10^−39^	Phosphorylates adenosine
Acetyl CoA thiolase	GB18952	−0.54	1.13×10^−19^	Converts acetyl CoA to acetoacetyl CoA
	GB12956	−3.58	1.00×10^−40^	
Hydroxymethylglutaryl-CoA synthase	GB12497	−0.15	0.35	Condenses Acetyl-CoA with acetoacetyl-CoA to HMG-CoA
Hydroxymethylglutaryl-CoA reductase	GB18242	−0.31	9.50×10^−5^	Reduces HMG-CoA to mevalonate
Mevalonate kinase	GB14701	−0.67	1.00×10^−7^	Phosphorylates mevalonate
Phosphomevalonate kinase-like protein	GB11982	−0.27	0.14	Phosphorylates phosphomevalonate
Diphosphomevalonate decarboxylase	GB16296	−0.83	1.19×10^−16^	Converts phosphomevalonate to isopentenyl diphosphate (IPP)
Isopentenyl diphosphate delta isomerase	GB44492	−0.55	1.57×10^−14^	Converts IPP to dimethylallyl pyrophosphate (DMAPP)
Farnesyl pyrophosphate synthase	GB18623	1.40	4.14×10^−61^	Formation of farnesyl disphosphate from condensation of IPP and DMAPP
	GB12385	−0.60	5.69×10^−12^	
	GB15337	1.22	3.15×10^−75^	
	GB18623	−1.76	2.28×10^−11^	
Citrate synthase	GB12573	−0.18	2.00×10^−3^	Citrate synthesis (mitochondria)
ATP citrate lyase	GB10992	0.19	5.28×10^−5^	Synthesis of acetyl CoA from citrate
Short-chain dehydrogenase/reductase	GB12522	1.87	7.20×10^−4^	Oxidation of farnesol to farnesal
GB14607	−1.20	5.48×10^−217^		
Juvenile hormone acid methyltransferase	GB10517	−5.76	1.00×10^−40^	Transfer of a methyl group to farnesoic acid
Methyl farnesoate epoxidase (CYP15A1)	GB15634	−3.52	1.00×10^−41^	Oxidation of methyl farnesoate to juvenile hormone III

Note, no genes within the JH pathway were predicted to be a miRNA target.

^*^Log Fold Change represents the expression level in workers relative to queens, as determined by high throughput sequencing. A negative value indicates a down-regulation in gene expression in workers relative to queens, a positive value indicates the opposite. FDR = False discovery rate adjusted P-value.

**Table 4:** An example of increased expression of enzymes associated with metobolic activity (citric acid cycle) in queens relative to workers.

Enzyme (Citric Acid Cycle)	GB Number	Log Fold Change*	FDR	Predicted Targeting miRNAs	Function
Citrate synthase 1, mitochondrial-like	GB52073	−0.18	2.00 × 10^−3^		Catalyses the formation of citrate from acetyl-CoA and oxaloacetate
ATP citrate lyase	GB54216	0.19	5.28 × 10^−5^		Catalytic conversion of citrate and CoA to acetly-CoA and oxaloacetate
Aconitate hydratase	GB43618	0.93	6.29 × 10^−57^	miR-316, miR-6063	Catalyses the isomerisation of citrate to isocitrate via cis-aconitate
Isocitrate dehydrogenase	GB45258	−1.23	2.74 × 10^−168^		Isomerisation of isocitrate to 2-oxoglutarate
	GB48527	−0.81	8.47 × 10^−26^		
2-oxoglutarate dehydrogenase, mitochondrial-like	GB50958	0.38	7.65 × 10^−7^	miR-279b	Enzyme complex that catalyses the conversion of 2-oxoglutarate to succinyl-CoA
Dihydrolipamide dehydrogenase 1	GB51335	−0.50	7.92 × 10^−11^		
Dihydrolipoyllysine-residue succinyltransferase	GB44430	0.66	2.15 × 10^−15^		
Succinyl coenzyme A synthetase alpha subunit	GB49632	−0.94	2.48 × 10^−126^		Catalyses the reversible reaction of succinyl-CoA to succinate
Succinate dehydrogenase [ubiquinone] flavoprotein subunit, mitochondrial-like	GB47042	0.47	0.10	miR-6056	Oxidation of succinate to fumarate, with the transfer of electrons from succinate to ubiquinone (coenzyme Q)
Fumarate hydratase, mitochondrial-like	GB51042	−0.62	2.60 × 10^−24^	miR-6059, miR-3741	Catalyses the conversion of fumarate to malate
Malate dehydrogenase, mitochondrial-like	GB54720	−1.21	2.00 × 10^−2^		Catalyses the conversion of malate to oxaloacetate
	GB42526	−1.69	1.00 × 10^−60^	miR-12	
Phosphoenolpyruvate carboxykinase	GB51494	−4.36	1.00 × 10^−60^		Catalyses the conversion of oxaloacetate (OAA) to phosphoenolpyruvate (PEP)
Dihydrolipoamide S-acetyltransferase	GB53566	−0.12	0.54	miR-3741, miR-6059, miR-3792	Catalyses the overall conversion (acetyl transfer) of pyruvate to acetyl-CoA
Pyruvate dehydrogenase (lipoamide) beta	GB55496	−0.26	2.00 × 10^−2^		
Pyruvate carboxylase, mitochondrial-like	GB40280	0.57	1.92 × 10^−43^	miR-315	Catalyses the carboxylation of pyruvate to oxaloacetate (OAA)
Dihydrolipoamide dehydrogenase 1	GB51335	−0.50	7.92 × 10^−11^	miR-279b	Oxidises dihydrolipoamide to lipoamide

^*^Log Fold Change represents the expression level in workers relative to queens, as determined by high throughput sequencing. A negative value indicates a down-regulation in gene expression in workers relative to queens, a positive value indicates the opposite. FDR = False discovery rate adjusted P-value.

**Table 5:** An example of gene enrichment in pathways associated with neuronal development (cholinergic synpase) in workers relative to queens.

Gene (Cholinergic Synpase)	GB Number	Log Fold Change*	FDR	Predicted Targeting miRNAs	Function
Acetylcholinesterase 1	GB43191	2.23	9.15 × 10^−24^	miR-6063, miR-6065, miR-3749, miR-6012, miR-3751	Hydrolysis of acetylcholine
Acetylcholinesterase 2	GB41856	2.21	1.80 × 10^−28^	miR-3718a, miR-3749, miR-3741, miR-3776, miR-3768, miR-3049-3p	
Voltage-dependent calcium channel type D subunit alpha-1	GB55480	2.23	5.29 × 10^−12^	miR-3741, miR-3739, miR-965-5p, miR-210, miR-3768	Mediate entry of calcium ions
Inositol 1,4,5,-tris-phosphate receptor	GB46583	0.39	7.96 × 10^−9^		Inositol triphosphate mediated calcium channel
Adenylate cyclase type 8	GB54593	0.39	2.34 × 10^−5^		Membrane-bound enzyme that catalyzes the formation of cAMP
Adenylate cyclase 3	GB45150	1.08	2.28 × 10^−3^		Membrane-associated enzyme that catalyzes the formation of cAMP
Phospholipase C	GB44174	−0.43	1.00 × 10^−4^	gi|323388982|ref|NC_007075.3|:4427403-4427428:-, gi|323388984|ref|NC_007073.3|:76047-76070: + , miR-3755, miR-6039, miR-210, miR-6012	Catalyzes the hydrolysis of phosphatidylinositol-4,5-bisphosphate to generate inositol 1,4,5-triphosphate (IP3) and diacylglycerol (DAG)
Phosphatidylinositol-4,5-bisphosphate 3-kinase catalytic subunit delta isoform	GB43074	1.72	4.69 × 10^−60^	miR-6056, miR-6059, miR-3730, gi|323388987|ref|NC_007070.3|:9617943-9617963:-, miR-3746, miR-3741	Phosphorylates phosphatidylinositol 4,5-bisphosphate to generate phosphatidylinositol 3,4,5-trisphosphate (PIP3)
Protein kinase C	GB43222	2.90	6.78 × 10^-46^	miR-3751, miR-6066, miR-3792, gi|323388987|ref|NC_007070.3|:9617943-9617963:-, miR-928, miR-3718b, gi|323388984|ref|NC_007073.3|:251491-251510: + , miR-3776, miR-3775, miR-3739, miR-6042, miR-965-5p, miR-6059, miR-3741, gi|323388978|ref|NC_007079.3|:1762825-1762846:-, miR-6056	Protein phosphorylation
Dual specificity mitogen-activated protein kinase kinase dSOR1	GB41067	0.76	3.01 × 10^−3^	miR-3741, miR-3777	Dual-specific protein kinase that sits down-stream of Raf in the MAPK pathway
Mitogen-activated protein kinase ERK-A	GB51503	0.10	2.58 × 10^−6^		Protein kinase that sits down-stream of Raf in the MAPK pathway
High-affinity choline transporter 1	GB43293	2.48	5.62 × 10^−7^	gi|323388982|ref|NC_007075.3|:15585205-15585227:-, miR-6056, gi|323388972|ref|NC_007085.3|:3190449-3190472:-, miR-6063, gi|323388987|ref|NC_007070.3|:9617943-9617963:-, miR-6059, gi|323388984|ref|NC_007073.3|:76047-76070:+	Imports choline from extracellular space
Guanine nucleotide-binding protein beta subunit 2	GB54181	1.63	1.84 × 10^−163^	miR-3049-5p, miR-6048, miR-6049, gi|323388976|ref|NC_007081.3|:2775657-2775678:-, miR-210, miR-3775, miR-3776, gi|323388987|ref|NC_007070.3|:1183784-1183805: + , gi|323388982|ref|NC_007075.3|:15585205-15585227:-, miR-6056, miR-3740, miR-3741, gi|323388982|ref|NC_007075.3|:15584822-15584844:-, miR-3751, miR-3717, gi|323388984|ref|NC_007073.3|:251491-251510: + , miR-3790	Involved in the recruitment, assembly and/or regulation of a variety of signaling molecules
Guanine nucleotide-binding protein G(o) subunit alpha	GB54642	1.34	2.92 × 10^−10^	miR-282, miR-6063	
Guanine nucleotide-binding protein G(q) subunit alpha	GB43282	1.52	7.91 × 10^−51^	miR-263a, miR-14	
Potassium voltage-gated channel subfamily KQT member 1	GB51854	0.42	5.97 × 10^−4^	gi|323388972|ref|NC_007085.3|:379513-379535: + , miR-3761, gi|323388984|ref|NC_007073.3|:76047-76070: + , miR-3741, gi|319965553|ref|NW_003378466.1|:4745-4762: + , miR-3717	Voltage-gated potassium channel, normally associated with caridiac cell repolarisation
cAMP-dependent protein kinase 3	GB48362	0.78	4.24 × 10^−5^		cAMP-dependent phopohrylation of target proteins
Calcium/calmodulin-dependent protein kinase II	GB49535	1.25	4.59 × 10^−22^	miR-3790, gi|323388979|ref|NC_007078.3|:10730925-10730949:+	Calcium/calmodulin-dependent serine/threonine protein kinase
Phosphotidylinositol 3 kinase 21B	GB42200	0.95	7.74 × 10^−22^		Phophorylation of the inositol ring of phosphatidylinositol
Muscarinic acetylcholine receptor	GB51689	0.94	1.56 × 10^−4^	miR-34	Cholinergic receptor family
Cyclic AMP response element-binding protein A	GB47941	0.62	6.97 × 10^−4^	miR-92a, miR-92b	Transcription factor

^*^Log Fold Change represents the expression level in workers relative to queens, as determined by high throughput sequencing. A negative value indicates a down-regulation in gene expression in workers relative to queens, a positive value indicates the opposite. FDR = False discovery rate adjusted P-value.
