# Supplementary material for: MicroRNAs in Honey Bee Caste Determination
Source: Sci Rep. 2016 Jan 7;6:18794. doi: 10.1038/srep18794 (PMC4704047; doi:10.1038/srep18794)
Supplement: Supplementary Information [file srep18794-s1.pdf]

MicroRNAs in Honey Bee Caste Determination

Regan Ashby[1,2], Sylvain Forêt[1]*, Iain Searle[1,3], Ryszard Maleszka[1]*

Supporting Information

Supplementary Figure S1: Distances between neighbouring miRNAs for (A) previously known, and (B) novel miRNAs.

Supplementary Table S1: Nucleotide sequence information and chromosomal locations of novel miRNAs

Supplementary Table S2: GO and KEGG gene set enrichment analyses for differentially expressed genes, miRNA targets, methylated genes and differentially methylated genes.

Tab 1.1: GO analysis for differentially expressed genes,

Tab 1.2: KEGG analysis for differentially expressed genes,

Tab 2.1: GO analysis for the predicted miRNA targets,

Tab 2.2: KEGG analysis for the predicted miRNA targets,

Tab 3.1: GO analysis for the predicted miRNA targets having a statistically significant Pearson correlation coefficient with their miRNA,

Tab 3.2: KEGG analysis for the predicted miRNA targets having a statistically significant Pearson correlation coefficient with their miRNA,

Tab 4.1: GO analysis for the methylated genes,

Tab 4.2: KEGG analysis for the methylated genes,

Tab 5.1: GO analysis for the differentially methylated genes,

Tab 5.2: KEGG analysis for the differentially methylated genes.

Supplementary Table S3: miRNA target prediction

Tab 1: Initial miRNA target prediction using the methods PITA, Miranda and RNAHybrid.

Tab 2: Refined miRNA target predictions based on a significant correlation between the expression of the miRNAs and their predicted transcript targets.

Supplementary Table S4: Overlapping GO terms enriched in miRNA target genes predicted by different methods.

Tab 1: Overlapping GO terms enriched in miRNA target genes predicted by PITA, Miranda and RNAHybrid.

Tab 2: Overlapping GO terms enriched in miRNA target genes predicted by PITA and Miranda.

Tab 3: Overlapping GO terms enriched in miRNA target genes predicted by Miranda and RNAHybrid.

Tab 4: Overlapping GO terms enriched in miRNA target genes predicted by PITA and RNAHybrid.

Supplementary Table S5: StemLoop Primers used for quantitative PCR analysis. Nucleotides within the 'miRNA Sequence' column labelled orange represent the targeting (complementary) sequence for the corresponding forward primer. Nucleotides labelled in red within the 'StemLoop Primer' column represent the universal probe binding site.

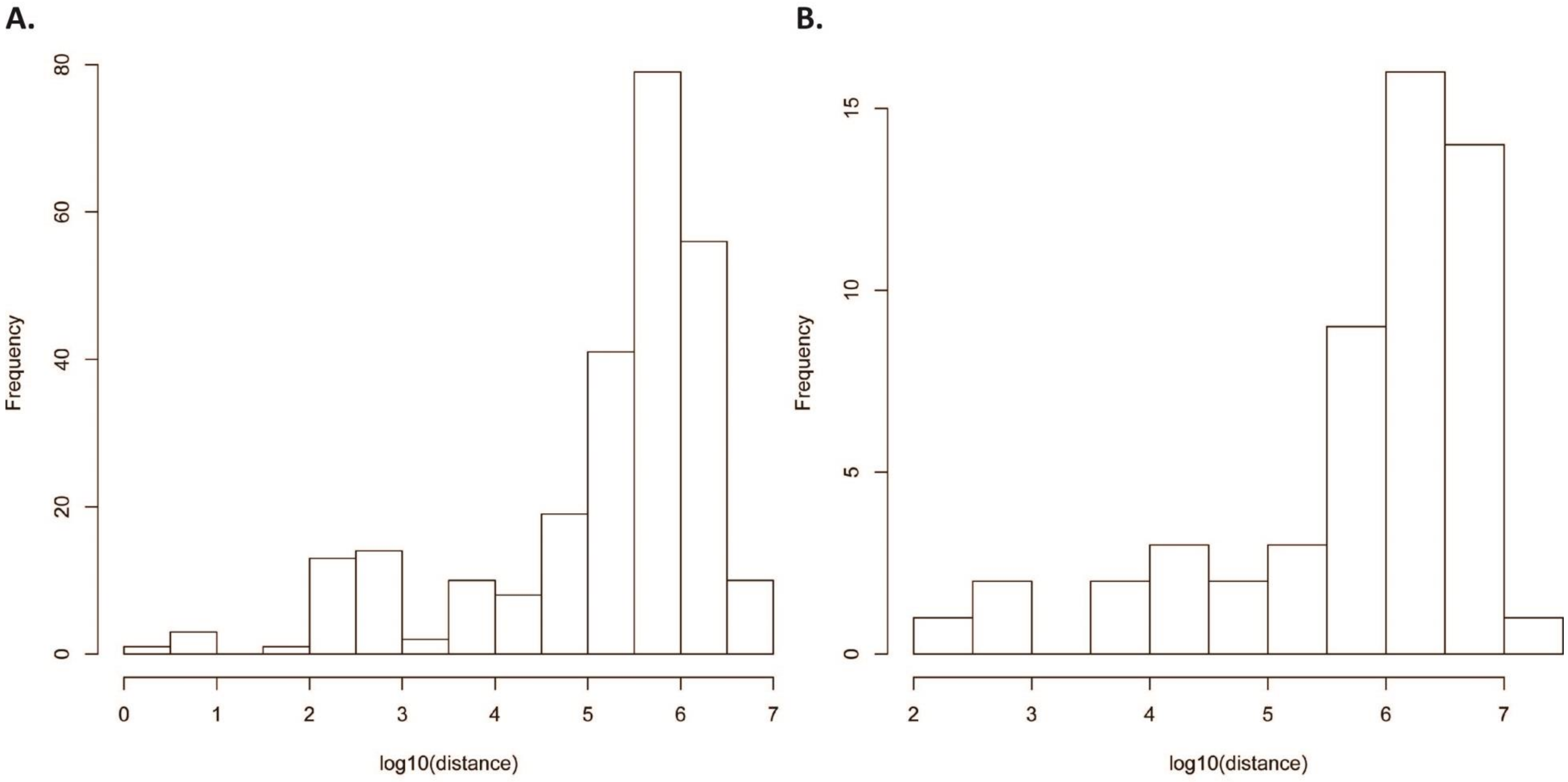

Supplementary Figure S1: Distances between neighbouring miRNAs for (A) previously known, and (B) novel miRNAs

No.	mirBase	miRNA Sequence	StemLoop Primer	Forward Primer
1	ame-Bantam	TGAGATCATTGTGAAAGCTGATT	GTTGGCTCTGGTGCAGGGTCCGAGGTATTCGCACCAGAGCCAACAATCAG	GCGCGTGTGAGATCATTGTG
2	ame-mir-1175	TGAGATTCACTCCTCCAACTTA	GTTGGCTCTGGTGCAGGGTCCGAGGTATTCGCACCAGAGCCAACTAAGTT	CCGCCTTGAGATTCACTCCT
3	ame-mir-13b	TATCACAGCCATTTTTGACGA	GTTGGCTCTGGTGCAGGGTCCGAGGTATTCGCACCAGAGCCAACTCGTCA	CCGCCCTATCACAGCCATTT
4	ame-mir-2	TCACAGCCAGCTTTGATGA	GTTGGCTCTGGTGCAGGGTCCGAGGTATTCGCACCAGAGCCAACTCATCA	CCGCCTATCACAGCCAGCTT
5	ame-mir-252a	ATAAGTACTAGTGCCGCAGGA	GTTGGCTCTGGTGCAGGGTCCGAGGTATTCGCACCAGAGCCAACTCCTGC	GCGGCGATAAGTACTAGTGC
7	ame-mir-276	TAGGAACTTCATACCGTGCTCTT	GTTGGCTCTGGTGCAGGGTCCGAGGTATTCGCACCAGAGCCAACAAGAGC	CGCCCGTAGGAACTTCATAC
8	ame-mir-2765-5p	TGGTAACTCCACCACCGTTGGCG	GTTGGCTCTGGTGCAGGGTCCGAGGTATTCGCACCAGAGCCAACCGCCAA	GCGTAGTGGTAACTCCACCA
9	ame-mir-283	AAATATCAGCTGGTAATTCTGGGA	GTTGGCTCTGGTGCAGGGTCCGAGGTATTCGCACCAGAGCCAACTCCCAG	CGCCCGAAATATCAGCTGGT
10	ame-mir-315	TTTTGATTGTTGCTCAGAAA	GTTGGCTCTGGTGCAGGGTCCGAGGTATTCGCACCAGAGCCAACTTTCTG	CGCCCGTTTTGATTGTTGCT
11	ame-mir-3715	TCGGTAAGCAGAGTATAA	GTTGGCTCTGGTGCAGGGTCCGAGGTATTCGCACCAGAGCCAACTTATAC	CGCCTCCATCGGTAAGCAGA
12	ame-mir-375	TTTGTTCGTTCGGCTCGAGTTA	GTTGGCTCTGGTGCAGGGTCCGAGGTATTCGCACCAGAGCCAACTAACTC	GGCGTGTTTGTTCGTTCGGC
13	ame-mir-6001-3p	TTCTCTTTGGTTGTTACCACT	GTTGGCTCTGGTGCAGGGTCCGAGGTATTCGCACCAGAGCCAACAGTGGT	GCGCGGGTTCTCTTTGGTTGT
14	ame-mir-71	TGAAAGACATGGGTAGTGA	GTTGGCTCTGGTGCAGGGTCCGAGGTATTCGCACCAGAGCCAACTCACTA	CGGGCACTGAAAGACATGGG
15	ame-mir-750	CCAGATCTAACTCTTCCAGCTCA	GTTGGCTCTGGTGCAGGGTCCGAGGTATTCGCACCAGAGCCAACTGAGCT	GCGGCTGCCAGATCTAACTC
16	ame-mir-87-1	GTGAGCAAAGTTTCAGGTGTGTG	GTTGGCTCTGGTGCAGGGTCCGAGGTATTCGCACCAGAGCCAACCACACA	CCGCCTGTGAGCAAAGTTTC
17	ame-mir-87-2	GTGAGCAAAGTTTCAGGTGTGTC	GTTGGCTCTGGTGCAGGGTCCGAGGTATTCGCACCAGAGCCAACGACACA	CCGCCTGTGAGCAAAGTTTC
18	ame-mir-let7	TGAGGTAGTAGGTTGTATAGTA	GTTGGCTCTGGTGCAGGGTCCGAGGTATTCGCACCAGAGCCAACTACTAT	CGCCGATGAGGTAGTAGGTT
20	ame-mir-263b (Control 1)	CTTGGCACTGGAAGAATTCACAGA	GTTGGCTCTGGTGCAGGGTCCGAGGTATTCGCACCAGAGCCAACTCTGTG	CGGGTTCTTGGCACTGGAAG
21	ame-mir-11 (Control 2)	CATCACAGGCAGAGTTCTAGTT	GTTGGCTCTGGTGCAGGGTCCGAGGTATTCGCACCAGAGCCAACAACTAG	GCGTACCCATCACAGGCAGA
22	Universal reverse primer			GTGCAGGGTCCGAGGT

Supplementary Table 5: StemLoop Primers used for quantitative PCR analysis. Nucleotides within the ‘miRNA Sequence’ column labelled orange represent the targeting (complementary) sequence for the corresponding forward primer. Nucleotides labelled in red within the ‘StemLoop Primer’ column represent the universal probe binding site.
